# Biodistribution and elimination kinetics of systemic Stx2 by the Stx2A and Stx2B subunit-specific human monoclonal antibodies in mice

**DOI:** 10.1186/1471-2172-13-27

**Published:** 2012-06-01

**Authors:** Abhineet Sheoran, Kwang-il Jeong, Jean Mukherjee, Anthony Wiffin, Pradeep Singh, Saul Tzipori

**Affiliations:** 1 Department of Biomedical Sciences, Cummings School of Veterinary Medicine, Tufts University, 200 Westboro Road Building 20, North Grafton, MA, USA; 2 Vaccine Research Center, 401 North Middletown Road Pfizer, Pearl River, NY, 10965, USA; 3 Department of Mathematics, Southeast Missouri State University, 401 North Middletown Road Pfizer, Cape Girardeau, MO, 63701, USA

**Keywords:** Shiga toxin, Radiolabel, Antibody, Toxin elimination, Toxin concentration, Pharmacokinetic, Human monoclonal antibody

## Abstract

**Background:**

Hemolytic uremic syndrome (HUS) leading to acute kidney failure, is a condition linked to the production of primarily Shiga toxin 2 (Stx2) by some *E. coli* serotypes. We have previously shown that Stx2 A subunit-specific human monoclonal antibody (HuMAb) 5C12, and B subunit-specific HuMAb 5H8 inhibit cultured cell death, and protect mice and piglets from fatal Stx2-intoxication. We have also shown that 5H8 blocks binding of Stx2 to its cell-surface receptor globotriaosyl ceramide (Gb_3_), whereas Stx2 when complexed with 5C12 binds Gb_3_ with higher affinity than Stx2. The mechanism by which 5C12 neutralizes Stx2 in vitro involves trapping of Stx2 in the recycling endosomes and releasing it into the extracellular environment. Because of the clinical implications associated with the formation of Stx2/antibody complexes and the potential for trapping and clearance through a severely damaged kidney associated with HUS, we investigated the likely site(s) of Stx2/antibody localization and clearance in intoxicated mice treated with antibody or placebo.

**Results:**

Mice were injected with radiolabeled Stx2 (^125^I-Stx2) 4 hours after administration of 5C12, 5H8, or phosphate buffered saline (PBS) and the sites of localization of labeled Stx2, were investigated 3, 24 and 48 hours later. The liver recorded statistically much higher concentrations of labeled Stx2 for groups receiving 5C12 and 5H8 antibodies after 3, 24 and 48 hours, as compared with the PBS group. In contrast, highest levels of labeled Stx2 were detected in the kidneys of the PBS group at all 3 sampling times. Mice receiving either of the two HuMAbs were fully protected against the lethal effect of Stx2, as compared with the fatal outcome of the control group.

**Conclusions:**

The results suggest that HuMAbs 5C12 and 5H8 promoted hepatic accumulation and presumably clearance of toxin/antibody complexes, significantly diverting Stx2 localization in the kidneys, the target of Stx2 and the cause of HUS. This is in contrast to the fatal outcome of the control group receiving PBS. The results also confirm earlier observations that both HuMAbs are highly and equally protective against Stx2 intoxication in mice.

## Background

Infection with Shiga toxin (Stx)-producing *Escherichia coli* (STEC) is the most significant cause of hemolytic uremic syndrome (HUS), the leading cause of acute renal failure in children [1-4]. Of the two antigenically distinct toxins, Stx1 and Stx2, Stx2 is more firmly linked with the development of HUS. Stx1 and Stx2 are similar in basic structure [5], binding specificity [5] and mode of action. Epidemiologic studies show that Stx2-producing strains are more frequently associated with HUS than strains that produce both Stx1 and Stx2; while Stx1 alone has rarely been associated with HUS [6-8].

Stx1 and Stx2 consist of an A-subunit monomer and a B-subunit pentamer [5,9,10]. The pentameric B subunit binds to its cell surface receptor globotriaosyl ceramide (Gb_3_; Galα1-4Galβ1-4glucosyl ceramide) [11,12]. Internalized Stx follows retrograde transport to the trans-Golgi network and subsequently to the endoplasmic reticulum and cytosol [13,14]. During this trafficking, the A subunit is nicked by the membrane-bound furin protease, generating a catalytically active N-terminal A1 fragment and a C-terminal A2 fragment; both fragments remain linked by a disulfide bond [13,15]. The disulfide bond is subsequently reduced, and the active A1 component is released. The released A1 fragment has N-glycosidase catalytic activity and it removes a specific adenine base from the 28S rRNA of the 60S ribosomal subunit [16,17]. Because this adenine base is on a loop of rRNA that is important for elongation factor binding, Stx is able to shut down the protein synthesis and cause cell death.

We have produced human monoclonal antibodies (HuMAbs) against Stx1 and Stx2, and evaluated them in animal models for their efficacy against systemic challenge with the toxins [18,19]. We selected 5C12, a Stx2 A subunit-specific HuMAb, based on its superior efficacy in protecting mice against lethal challenge with Stx2 and Stx2 variants [20] for preclinical evaluation in the piglet diarrhea model challenged orally with STEC. This antibody protected piglets against Stx2-induced fatal neurological symptoms, even when administered well after the onset of diarrhea following oral STEC challenge (48 hours post-challenge) [21]. In this model, diarrheal symptoms precede systemic complications associated with Stx2 uptake from the gut, as is observed in children. Similarly, Stx2 B subunit-specific HuMAb 5 H8 also protects piglets [18] and mice against Stx2 intoxication [18,21].

While these HuMAbs completely protect healthy piglets and mice from Stx2-mediated death, there remains a concern among nephrologists for the potential formation of immune Stx2/antibody complexes in a severely damaged kidney of patients with HUS. In the mouse model, the kidneys are the major target organ of Stx2-intoxication. In this model, Stx2 causes apoptosis of medullary and cortical tubular cells in the kidneys, and leads to renal failure due to the loss of functioning collecting ducts [22]. In the present experiments we investigated the likely site of Stx2/antibody localization and clearance using the mouse model of systemic intoxication treated with these potentially therapeutic antibodies.

## Methods

### Stx2

Stx2, purified from *E. coli* C600W as described previously [23], was obtained from Phoenix Laboratory (Tufts University-NEMC Microbial Products & Services Facility). The toxin was dissolved at 500 μg/ml in phosphate buffered saline (PBS), aliquoted and stored at −20°C.

### Stx2-specific HuMAbs

Production of Stx2A-specific 5C12 and Stx2B-specific 5H8 HuMAbs have been described elsewhere [18,21]. These antibodies were purified by protein A affinity chromatography, dialyzed against PBS, quantified by UV spectrophotometry (ND-1000 Spectrophotometer, Nanodrop), aliquoted, and stored at −20°C.

### Iodination of Stx2

Purified Stx2 was iodinated by a modification of the chloramine T method [24,25]. Briefly, one millicurie of carrier-free Na^125^I (PerkinElmer, Billerica, MA) was added to 20 to 30 μg of Stx2 in 100 μl of PBS. Then, 20 μl of chloramine T (2.5 mg/ml in PBS; GFS Chemicals, Columbus, OH) was added, and incubated for 20 seconds. The iodination was terminated by adding 20 μl of sodium metabisulfite (5 mg/ml in distilled water; Sigma, St. Louis, MO). Ten μl of potassium iodide (20 mg/ml in PBS; Sigma) was subsequently added to scavenge the free iodine. The iodinated Stx2 (^125^I-Stx2) was separated from free ^125^I by filtration through a polyacrylamide column (D-salt^TM^ columns; Pierce, Rockford, IL).

### Biodistribution of ^125^I-Stx2, and ^125^I-Stx2/HuMAb complexes

The mouse toxicity model [21] was used to study biodistribution of ^125^I-Stx2, and ^125^I-Stx2/HuMAb complexes. Four-week-old female C57BL/6 mice weighing 18–22 g (Charles River Laboratories) were injected intraperitoneally (IP) with PBS or 30 μg/mouse of a Stx2-specific HuMAb (5C12 or 5H8). After 4 hr, mice were administered intravenously (IV) with 100 ng/mouse (in 100 μl of PBS) of ^125^I-Stx2 as a single bolus dose. The ^125^I-Stx2 dose of 100 ng/mouse is 4 times the LD100 dose [21]. An isotype control was not included in the current experiments as we have consistently found no difference between isotype control and PBS groups [21]. The isotype control antibody does not interact with the toxin and mice die within 3 days of either PBS or isotype control antibody administration [21]. Similarly, piglets also succumb to Stx2-mediated systemic complication following either PBS or isotype control antibody administration [21]. A group of 3 mice from each treatment group (5C12 or 5H8 or PBS) was euthanized at 3 h, 1 and 2 days post-^125^I-Stx2 injection. Blood was collected from these mice before euthanasia, and tissues and urine were collected after euthanasia. The fluids and tissues were analyzed for radioactivity by a gamma counter (1470 Wallac wizard, Global Medical Instrumentation, Inc., Ramsey, MN). A group of 5 mice injected IV with 100 ng of ^125^I-BSA was used as a control to the radiolabeled toxin injected group.

Stx2 was labeled again with ^125^I and the experiment repeated once to obtain 6 mice per treatment group. The data were presented as a percentage of injected dose of radioactive toxin (^125^I-Stx2) per gram (% ID/g) of an organ tissue, or per ml (% ID/ml) of a fluid. The% ID/g or% ID/ml was calculated by the formula: (counts per minute or CPM per ml or per gram / injected CPM) x 100.

### Biological activity of the radiolabeled Stx2

The mouse toxicity model was also used to ensure that radiolabeled Stx2 was biologically active and that Stx2-specific HuMAbs neutralized the toxin activity. Briefly, 30 μg/mouse of a Stx2-specific HuMAb, or PBS, was injected IP 4 hr prior to IV administration of 100 ng ^125^I-Stx2/mouse. Mice were observed 3 or more times daily for clinical signs and survival until 11 day post-toxin administration.

All animal studies described in the above 2 sections were performed in accordance with the guidelines of the Institutional Animal Care and Use Committee, Tufts University.

### Statistical analysis

For statistical analysis of total radioactivity remaining in the body following 3 h, 1 and 2 days of ^125^I-Stx2 injection in different treatment groups, the pair wise comparison was done using Student t-test and Wilcoxon rank test. For statistical analysis of radioactivity in each organ at a particular time point (3 h, 1 day or 2 days post-^125^I-Stx2 injection), nonparametric Kruskal-Wallis and Wilcoxon rank tests were performed to evaluate significant differences among the four treatment groups. The analyses were performed using statistical software (SAS 9.2). Resulting p-values of less than 0.05 were considered significant.

## Results

### Biological activity of radiolabeled (^125^I-Stx2) and neutralization of ^125^I-Stx2 by Stx2-specific HuMAbs

**Figure 1:**
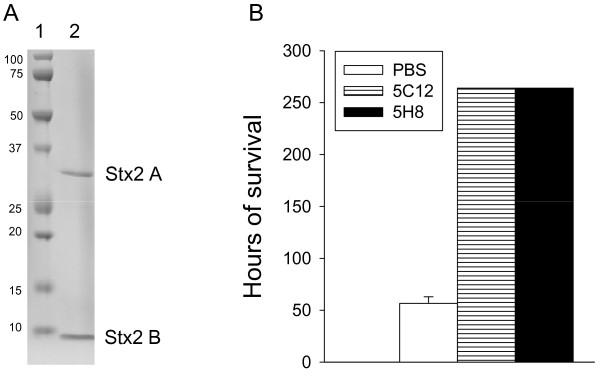
**(A): 12% SDS PAGE gel stained with Coomassie blue shows Stx2 preparation was pure as it contained only A and B subunits of Stx2 (lane 2); the molecular weight markers (in thousands) were in lane 1. (B):** Survival of mice given intraperitoneally PBS or 30 μg of HuMAbs 5C12 or 5H8 followed 4 hours later with intravenous administration of 100 ng of ^125^I-Stx2. Mice in PBS group died between 57–64 h or 2.3-2.6 days after ^125^I-Stx2 injection. Both 5C12 and 5H8 protected 100% of mice until the end of the experiment (264 h or 11 days).

Purified Stx2 consisted of only A and B subunits (Figure 1A). ^125^I-Stx2 was biologically active as none of the mice survived ^125^I-Stx2 challenge in the PBS group (Figure 1B). They either died or became severely ill and were euthanized 57–64 h or 2.3-2.6 days following toxin administration (Figure 1B). All Stx2-specific HuMAbs neutralized the lethal biological activity of ^125^I-Stx2 as all mice in HuMAb groups survived the ^125^I-Stx2 challenge until the end of the experiment (264 h or 11 day post-toxin challenge) (Figure 1B).

### Distribution of ^125^I-Stx2, and ^125^I-Stx2/HuMAb complexes in blood

In the control ^125^I-BSA group, 98% of ^125^I-BSA was eliminated from the body, and most of the remaining 2% ^125^I-BSA was localized in urine and blood 1 day post-^125^I-BSA administration (results not shown).

**Figure 2:**
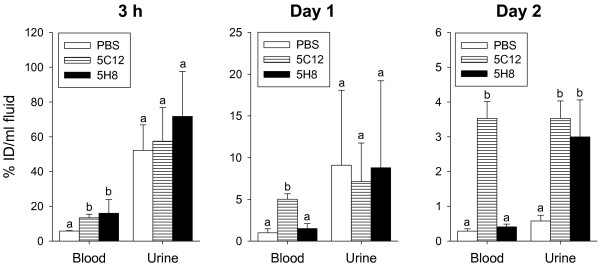
**Distribution of**^**125**^**I-Stx2 in body fluids following 3 h, 1 and 2 days of**^**125**^**I-Stx2 injection.** Mice were given intraperitoneally PBS or 30 μg of HuMAbs 5C12 or 5H8 followed 4 hours later with intravenous administration of 100 ng of ^125^I-Stx2. Distribution of ^125^I-Stx2 in body fluids was determined following 3 h, day 1 and day 2 of ^125^I-Stx2 injection, and expressed as “% injected dose (ID)/ml fluid”. Bars with different letters (a and b) within a body fluid at a particular sampling time point indicate differences are statistically significant (*p* < 0.05) among those treatment groups. Bars with same letter within a body fluid at a particular sampling time point indicate differences are not statistically significant among those treatment groups.

At 3 h post-^125^I-Stx2 administration, the toxin localized significantly more in blood of groups receiving HuMAbs 5C12 or 5H8 than the PBS group (Figure 2). However, the concentration of the toxin between the HuMAb groups did not differ significantly at this time point.

On days 1 and 2, the toxin localized significantly more in blood of groups receiving HuMAbs 5C12 than the PBS and 5H8 groups (Figure 2). The toxin concentration did not differ significantly between 5H8 and PBS. On these days, the relative mean toxin concentration in blood in each group was: 5C12 > 5H8 > PBS (Figure 2).

### Distribution of ^125^I-Stx2, and ^125^I-Stx2/HuMAb complexes in urine

Excretion of radioactivity in urine did not differ significantly between treatment groups at 3 h and 1 day after ^125^I-Stx2 administration (Figure 2). On day 2, the radioactivity localized significantly more in the urine of HuMAb groups than the PBS group (Figure 2).

### Distribution of ^125^I-Stx2, and ^125^I-Stx2/HuMAb complexes in tissues

**Figure 3:**
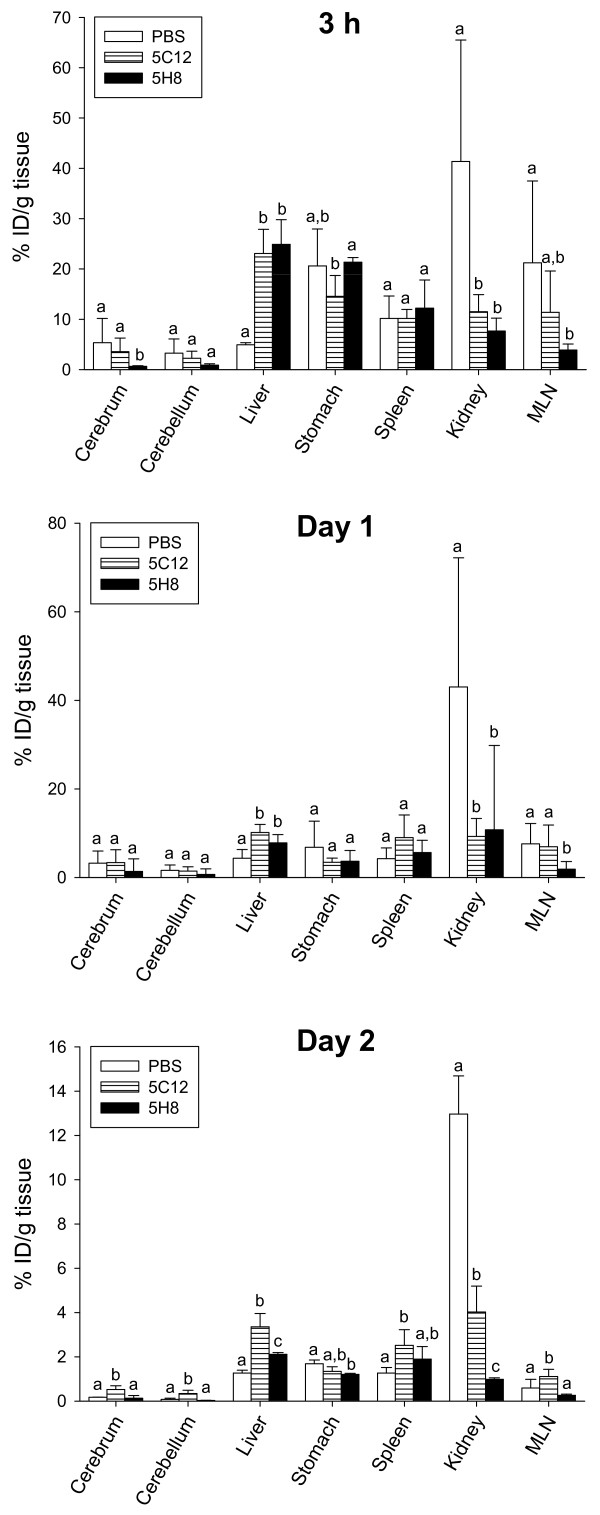
**Distribution of**^**125**^**I-Stx2 in tissues following 3 h, 1 and 2 days of**^**125**^**I-Stx2 injection.** Mice were given intraperitoneally PBS or 30 μg of HuMAbs 5C12 or 5H8 followed 4 hours later with intravenous administration of 100 ng of ^125^I-Stx2. Distribution of ^125^I-Stx2 in body tissues was determined following 3 h, day 1 and day 2 of ^125^I-Stx2 injection, and expressed as “% injected dose (ID)/g tissue”. Bars with different letters (a-c) within a tissue indicate differences are statistically significant (*p* < 0.05) among those treatment groups. Bars with same letter within a tissue indicate differences are not statistically significant among those treatment groups.

At 3 h, the toxin concentration in the PBS group was significantly different from both 5C12 and 5H8 antibody treatment groups in only two organs, the kidneys and the liver (Figure 3 and Table 1). In the cerebrum, the toxin concentration in the 5H8 group was significantly different from the PBS and 5C12 groups, and in the stomach, the toxin concentration in the 5H8 group was significantly different from the 5C12 group but not from the PBS group (Figure 3). In the mesenteric lymph nodes (MLN), the toxin concentration in the 5H8 group differed significantly from the PBS group but not from the 5C12 group (Figure 3). The toxin concentration in any of the remaining organs did not differ significantly between any treatment groups (Figure 3, Additional file 1: Table S1). Based on the numerical mean radioactivity concentration, highest concentrations were found in the kidneys (41%), liver (23%) and liver (25%) of PBS, 5C12 and 5H8 groups, respectively (Figure 3 and Table 1). While the toxin concentration in the kidneys was significantly greater in the PBS group than the HuMAb groups, it did not differ significantly among kidneys of the 5C12 and 5H8 groups. The toxin concentration in the liver was significantly higher in the 5C12 and 5H8 groups than in the PBS group but did not differ significantly between the 5C12 and 5H8 groups.

**Table 1:** **Distribution of**^**125**^**I-Stx2 in the kidney and liver following 3 h, 1 day and 2 days of**^**125**^**I-Stx2 injection**^**1**^

Sampling time	Tissue	PBS	5C12	5H8
3 h	Kidney	41±24.1^a^	11±3.4^b^	8±2.6^b^
	Liver	5±0.4^a^	23±4.8^b^	25±4.9^b^
Day 1	Kidney	43±29.1^a^	9±4.0 ^b^	11±19.1^b^
	Liver	4±1.9^a^	10±1.8^b^	8±1.9^b^
Day 2	Kidney	13±1.7^a^	4±1.2^b^	1±0.^c^
	Liver	1±0.1^a^	3±0.6^b^	2±0.1^c^

^1^Distribution of ^125^I-Stx2 in the kidney and the liver following 3 h, 1 day and 2 days of ^125^I-Stx2 injection. Mice were given intraperitoneally PBS or 30 μg of HuMAbs 5C12 or 5H8 followed 4 hours later with intravenous administration of 100 ng of ^125^I-Stx2. Distribution of ^125^I-Stx2 in the kidney and the liver was determined following 3 h, 1 day and 2 days of ^125^I-Stx2 injection, and expressed as “% injected dose (ID)/g tissue”.

^a-c^ Values with different letters (superscripts ‘a’, ‘b’ and ‘c’) within a tissue indicate differences are statistically significant (*p* < 0.05) among those treatment groups. Values with same letter superscript within a tissue indicate differences are not statistically significant among those treatment groups.

On day 1, the toxin concentration in the PBS group again differed significantly from both 5C12 and 5H8 antibody treatment groups in only two organs, the kidneys and the liver (Figure 3 and Table 1). In the MLN, the toxin concentration in the 5H8 group differed significantly from the PBS and 5C12 groups (Figure 3), and in the heart, the toxin concentration in the PBS group differed significantly from the 5C12 group but not from the 5H8 group (Additional file 1: Table S1). The toxin concentration in any of the remaining organs did not differ significantly between any treatment groups (Figure 3, Additional file 1: Table S1). Maximal levels of Stx2 localized in the kidneys of PBS (43%) group, in the kidneys (11%) and liver (8%) of 5H8 group, and in the liver, spleen and kidneys (~10%) of 5C12 group (Figure 3 and Table 1). Liver was a major organ of toxin accumulation in 5H8 and 5C12 groups but not in PBS (4%) group. The toxin concentration in the kidneys of the PBS group was significantly greater than in the kidneys of the 5C12 and 5H8 groups. The toxin concentration in the liver of the PBS was significantly lower than that of the 5C12 and 5H8 groups. The toxin concentration in the liver and kidneys did not differ significantly between the 5C12 and 5H8 groups.

On day 2, based on the numerical means, the kidneys were the organ where maximum toxin accumulated in the PBS (13%) group, whereas maximal levels of the toxin in the 5H8 group localized in the liver and spleen (2%), and in the 5C12 group in the liver, kidneys and spleen (3-4%) (Figure 3 and Table 1). Significantly more toxin localized in the livers of the 5C12 and 5H8 groups than the liver of PBS group. The toxin was barely detectable in cerebrum and cerebellum (Figure 3), and other organs (Additional file 1: Table S1).

## Discussion

The purpose of this investigation was to establish the site(s) of Stx2 accumulation in the intoxicated host, and whether administration of highly protective antibodies impacts this outcome. In addition, the site of accumulation of Stx2/antibody complexes in patients with HUS is of particular concern to nephrologists, should the site be the kidneys leading to further renal dysfunction. The results of the present study suggest that HuMAbs 5C12 and 5H8 facilitated hepatic clearance of Stx2 as maximal levels of radioactivity in these groups were present in the liver at 3 h after ^125^I-Stx2 administration. The liver was also a major organ of radioactivity accumulation in these 2 groups at day 1 and day 2 sampling times, largely sparing the kidney. In contrast, kidneys were the major target organ of ^125^I-Stx2 accumulation in the PBS group at all 3 sampling times, indicating that rapid hepatic elimination of Stx2 did not occur in this group. Other studies have previously shown that soluble immune complexes are cleared primarily by the liver through uptake into Kupffer cells [26-28].

The results of the present study show that accumulation of Stx2 in the kidneys is significantly blocked by the HuMAbs 5C12 and 5H8 as radioactivity accumulated in the kidneys of these two groups at much lower levels than the kidneys of the PBS group. As all mice in the 5C12 and 5H8 groups did not show any clinical signs of Stx2-intoxication and survived, the renal presence of Stx2 in these mice appear to have had minimal clinical impact. In contrast, all mice in the PBS group succumbed to Stx2intoxication, and toxin presence in the kidneys of this group may have contributed to their deaths. Stx2 in the kidneys of mice is toxic and causes apoptosis of medullary and cortical tubular cells, and renal failure due to the loss of functioning collecting ducts [22]. These results suggest that Stx2/5C12 or Stx2/5H8 complexes are less likely to accumulate in the kidneys in patients with HUS, and less likely to exacerbate the condition further.

Gb3 expression on neuronal cells in mouse central nervous system and its binding with Stx2 has recently been shown [29], which also explains earlier observations of Stx2 localization in the spinal cord [30]. In the present study, significantly more toxin was measured in the cerebrum but not cerebellum of the PBS and 5C12 groups than in the 5H8 group at the 3 h sampling time. This suggests that toxin can bind to the cerebrum but not the cerebellum, and that 5H8 but not 5C12 can block this binding. Significant differences in toxin accumulation in cerebrum or cerebellum did not occur on day 1 or day 2 in any of the groups. Note however, that in the present study, cerebral toxin accumulation in PBS and 5C12 groups was much less than the accumulation of toxin in the kidney, a well known target of Stx2. The spinal cord was not included in the present study.

The stomach was one of the major organs of radioactivity accumulation (especially at 3 h sampling time) in all treatment groups. The stomach has previously been shown to accumulate radioactivity in mice injected with radiolabeled Stx2 [30]. Presumably, sequestration of the released radioiodide in the stomach may have occurred due to the dehalogenation effect [31].

Rutjes et al. [30] reported that the highest concentrations of Stx2 localized in the nasal turbinates followed by the lungs and kidneys. Stx2-specific antibodies were not used in that study [30]. The lungs, but not the nasal turbinates were included in the present study, and with regards to toxin concentration, they were the fourth highest in the PBS and 5H8 groups, and the sixth highest in 5C12 group at 3 h sampling time (Figure 3, Additional file 1: Table S1). As the previous [30] and the present studies used radioiodinated Stx2 and the IV route of toxin administration, the discrepancy between the two investigations may be related to sampling time after toxin administration (1 h vs. 3 h in our study), and/or the strain of mouse used (Balb/c vs. C57BL/6 in our study). Nevertheless, the kidney was a major target organ for Stx2 in both studies [30]. Stx2 is known to have a propensity for lymphoid cells [32-34] as is shown by binding of the toxin within MLN in the present study.

## Conclusions

The results of the present study suggest that the interaction of Stx2 with HuMAbs 5C12 and 5H8 inhibited accumulation of the toxin in the kidney, the major target organ of Stx2-intoxication, and lead instead to rapid accumulation of Stx2 in the liver, an organ known to clear soluble immune complexes in vivo [26-28].

## Abbreviations

Stx2: Shiga toxin 2; HuMAb: Human monoclonal antibody; Gb3: Globotriaosyl ceramide; I-Stx2: Radiolabeled Stx2; PBS: Phosphate buffered saline; STEC: Shiga toxin-producing Escherichia coli; HUS: Hemolytic uremic syndrome; % ID/g: Percentage of injected dose of radioactive toxin per gram of an organ tissue; % ID/ml: Percentage of injected dose of radioactive toxin per ml of a fluid; CPM: Counts per minute; MLN: Mesenteric lymph nodes.

## Competing interests

The authors have declared that no competing interests of any kind exist.

## Authors’ contributions

AS conceived and designed the experiments, and contributed to all aspects of this research; KJ, JM and AW performed the experiments; AS and PS analyzed the data; AS wrote the manuscript; ST and JM contributed to writing the manuscript, and KJ helped in addressing the reviewers’ comments. All authors read and approve the final manuscript.

## Supplementary Material

Additional file 1 **Table S1. **Distribution of ^125^I-Stx2 in body fluids and tissues following 3 h, 1 day and 2 days of ^125^I-Stx2 injection.^1^ Click here for file
