# Supplementary material for: Biodistribution and elimination kinetics of systemic Stx2 by the Stx2A and Stx2B subunit-specific human monoclonal antibodies in mice
Source: BMC Immunol. 2012 Jun 1;13:27. doi: 10.1186/1471-2172-13-27 (PMC3436740; doi:10.1186/1471-2172-13-27)
Supplement: Additional file 1 — Table S1. Distribution of 125I-Stx2 in body fluids and tissues following 3 h, 1 day and 2 days of 125I-Stx2 injection.1 [file 1471-2172-13-27-S1.doc]

Supplementary Table 1. Distribution of ^125^I-Stx2 in body fluids and tissues following 3 h, 1 day and 2 days of ^125^I-Stx2 injection.^1^

| Sampling time | Body fluid/tissue | PBS | 5C12 | 5H8 |
| --- | --- | --- | --- | --- |
|  | Thymus | 2±1.2 ^a^ | 2±0.5 ^a^ | 2±0.6 ^a^ |
|  | Lungs | 12±7.6 ^a^ | 7±1.2 ^a^ | 8±3.8 ^a^ |
| 3 h | Heart | 4±0.6 ^a^ | 5±0.6 ^a^ | 5±2.2 ^a^ |
|  | Small intestine | 4±16.2 ^a^ | 3±0.2 ^a^ | 4±0.7 ^a^ |
|  | Large intestine | 4±11.5 ^a^ | 3±0.2 ^a^ | 3±0.3 ^a^ |
|  | Pancreas | 6±1.9 ^a^ | 5±1.1 ^a^ | 4±0.2 ^a^ |
|  | Thymus | 1±0.7 ^a^ | 1±0.9 ^a^ | 1±0.2 ^a^ |
|  | Lungs | 3±2.2 ^a^ | 3±0.8 ^a^ | 2±2.1 ^a^ |
| Day 1 | Heart | 1±0.3 ^a^ | 2±0.3 ^b^ | 1±0.7 ^a,b^ |
|  | Small intestine | 1±0.5 ^a^ | 1±0.3 ^a^ | 1±0.3 ^a^ |
|  | Large intestine | 1±0.8 ^a^ | 1±0.2 ^a^ | 1±0.5 ^a^ |
|  | Pancreas | 2±2.1 ^a^ | 2±1.1 ^a^ | 1±0.3 ^a^ |
|  | Thymus | 0±0.0 ^a^ | 1±0.1 ^b^ | 0±0.0 ^c^ |
|  | Lungs | 1±0.1 ^a^ | 1±0.2 ^b^ | 0±0.1 ^c^ |
| Day 2 | Heart | 0±0.2 ^a^ | 1±0.3 ^b^ | 0±0.0 ^a^ |
|  | Small intestine | 0±0.0 ^a^ | 0±0.1 ^b^ | 0±0.0 ^a^ |
|  | Large intestine | 0±0.1 ^a^ | 0±0.0 ^a^ | 0±0.0 ^a^ |
|  | Pancreas | 0±0.1 ^a^ | 1±0.1 ^b^ | 0±0.0 ^c^ |

^1^Distribution of ^125^I-Stx2 in tissues following 3 h, 1 day and 2 days of ^125^I-Stx2 injection. Mice were given intraperitoneally PBS or 30 μg of HuMAbs 5C12 or 5H8 followed 4 hours later with intravenous administration of 100 ng of ^125^I-Stx2. Distribution of ^125^I-Stx2 in body tissues was determined following 3 h, 1 day and 2 days of ^125^I-Stx2 injection, and expressed as “% injected dose (ID)/g tissue”.

^a-c^ Values with different letters (superscripts ‘a’, ‘b’ and ‘c’) within a tissue indicate differences are statistically significant (*p* < 0.05) among those treatment groups. Values with same letter superscript within a tissue indicate differences are not statistically significant among those treatment groups.
